# DNA-Mediated Self-Assembly of Artificial Vesicles

**DOI:** 10.1371/journal.pone.0009886

**Published:** 2010-03-26

**Authors:** Maik Hadorn, Peter Eggenberger Hotz

**Affiliations:** 1 Artificial Intelligence Laboratory, Department of Informatics, University of Zurich, Zurich, Switzerland; 2 The Mærsk Mc-Kinney Møller Institute, University of Southern Denmark, Odense, Denmark; University of Helsinki, Finland

## Abstract

**Background:**

Although multicompartment systems made of single unilamellar vesicles offer the potential to outperform single compartment systems widely used in analytic, synthetic, and medical applications, their use has remained marginal to date. On the one hand, this can be attributed to the binary character of the majority of the current tethering protocols that impedes the implementation of real *multi*component or *multi*functional systems. On the other hand, the few tethering protocols theoretically providing *multi*compartment systems composed of several distinct vesicle populations suffer from the readjustment of the vesicle formation procedure as well as from the loss of specificity of the linking mechanism over time.

**Methodology/Principal Findings:**

In previous studies, we presented implementations of *multi*compartment systems and resolved the readjustment of the vesicle formation procedure as well as the loss of specificity by using linkers consisting of biotinylated DNA single strands that were anchored to phospholipid-grafted biotinylated PEG tethers via streptavidin as a connector. The systematic analysis presented herein provides evidences for the incorporation of phospholipid-grafted biotinylated PEG tethers to the vesicle membrane during vesicle formation, providing specific anchoring sites for the streptavidin loading of the vesicle membrane. Furthermore, DNA-mediated vesicle-vesicle self-assembly was found to be sequence-dependent and to depend on the presence of monovalent salts.

**Conclusions/Significance:**

This study provides a solid basis for the implementation of multi-vesicle assemblies that may affect at least three distinct domains. (i) Analysis. Starting with a minimal system, the complexity of a bottom-up system is increased gradually facilitating the understanding of the components and their interaction. (ii) Synthesis. Consecutive reactions may be implemented in networks of vesicles that outperform current single compartment bioreactors in versatility and productivity. (iii) Personalized medicine. Transport and targeting of long-lived, pharmacologically inert prodrugs and their conversion to short-lived, active drug molecules directly at the site of action may be accomplished if multi-vesicle assemblies of predefined architecture are used.

## Introduction

Artificial vesicles feature an aqueous compartment separated from an aqueous surrounding by a closed membrane that is almost impermeable for hydrophilic substances. Like cell membranes, vesicle membranes consist of amphiphilic phospholipids that link a hydrophilic head and a lipophilic tail. All tails pointing towards the center of the membrane resulting in a two-layered sheet (a bilayer). This molecular arrangement excludes water from the center of the sheet thereby eliminating entropic unfavorable contacts between water and the lipophilic ( =  hydrophobic) tails. The lipid bilayer organizes processes by compartmentalizing them and provides inherent self-repair characteristics due to lateral mobility of its phospholipids [1].

As a result of the analogy to natural systems and the compositional simplicity, artificial vesicles are the most studied systems among biomimetic structures [2] providing a bottom-up procedure in the analysis of biological processes [3]–[5]. In addition, vesicles are applied in synthetics where they are used both as mini-laboratories to study confined chemical reactions under biologically relevant conditions [6] and as bioreactors [7]–[9]. Their ability to control confinement, transport, and manipulation of chemical cargo is used in vesicular drug delivery systems [10]–[12]. Single unilamellar vesicles are used essentially in analytic, synthetic, and medical applications. In contrast, multicompartment systems offer a division of different membrane functions (confinement, biocompatibility, cargo release, targeting, protection) among membranes of distinct compositions and dimensions. Specific chemical reactions can be segregated for the purposes of increased controllability, observability, stability, and biochemical efficiency by restricted dissemination and efficient storage of reactants, and/or reaction products. Thus, tethered multi-vesicle systems have been realized in both bioreactor [13], [14] and cosmetic applications [15] and proposed as multicomponent or multifunctional drug delivery systems [16]–[19]. The authors already discussed potential applications of multi-vesicle systems in personalized drug delivery [20] and as real-world testbeds of results observed *in silico* [21].

Current tethering protocols of multi-vesicle systems are based either on electrostatic or donor-acceptor interactions [22]–[31]. Due to the binary character of these linking mechanisms real *multi*component or *multi*functional systems are hardly feasible. The programmable self-assembly of superstructures composed of *n* distinct entities with high degrees of complexity [32] has attracted significant attention in nanotechnological applications [33]–[36]. Since single stranded DNA (ssDNA) offers a multitude of distinct linkers, high specificity of binding between complementary sequences, and a digital nature of DNA base coding, it represents an ideal candidate for the implementation of multi-vesicle assemblies of programmable composition and spatial arrangement. DNA single strands were therefore introduced as crosslinking agents to induce the assembly of complementary monohomophilic hard sphere [34], [37]–[39] or vesicle [40], [41] colloids, to induce programmable fusion of vesicles [41], [42], or to spontaneously and specifically link vesicles to surface supported membranes [40], [41], [43]–[46]. However, current DNA-mediated linking mechanisms suffer from two shortcomings. In most cases, linkers are composed of ssDNA covalently linked to cholesterol [40], [43], [45], [46] or to lipids [41], [44]. Single cholesterol-tagged ssDNA (monocholesterol ssDNA) spontaneously leaves the lipid bilayer and incorporates randomly into (other) lipid bilayers [40], [47]. Thus, the specificity of the linking system is lost over time. Although this problem can be solved by using two anchors per ssDNA (e.g. bicholesterol ssDNA) [47] a second drawback remains intrinsic to the molecular architecture of the linkers. The partition coefficient of amphiphilic linkers is affected by the characteristics of their hydrophilic (ssDNA) and hydrophobic (membrane anchors) components. Thus, vesicle formation and/or composition have to be readjusted anew every time the characteristics (e.g. length of ssDNA) of the linkers are changed.

**Figure 1:**
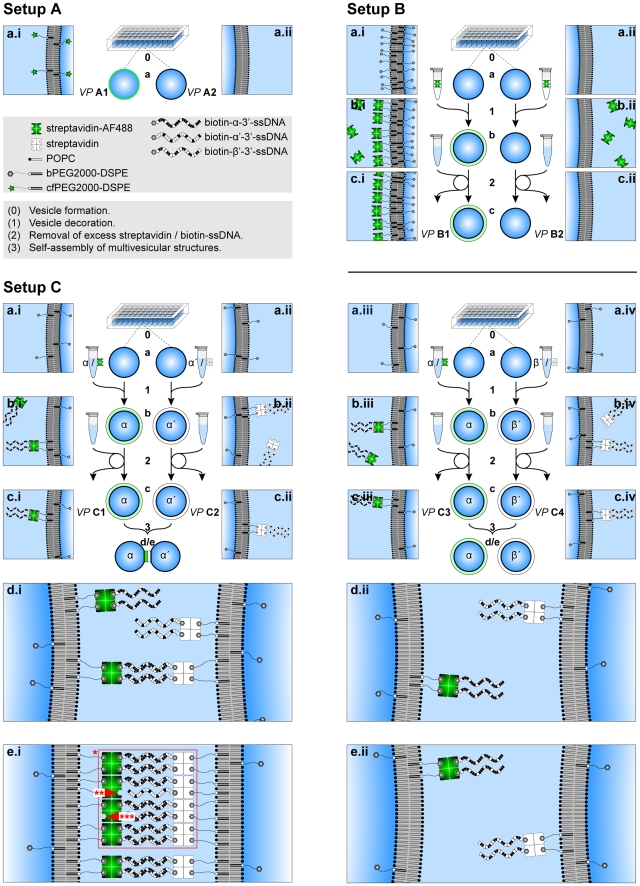
Schematic representation of experimental setups A, B, and C. Numbers (0–3) indicate processes and small letters (a–e) indicate states. (Setup A) The incorporation of phospholipid-grafted PEG tethers into the vesicle membrane is analyzed. Vesicle populations (VPs) differ in the presence (VP A1) and absence (VP A2) of phospholipid-grafted fluorescently labeled PEG tethers (cfPEG2000-DSPE) during vesicle formation. (Setup B) To settle the specificity of membrane loading with streptavidin depending on the presence of anchoring sites, phospholipid-grafted biotinylated PEG tethers (bPEG2000-DSPE) are either present (VP B1) or absent (VP B2) during vesicle formation. Both VPs are subsequently incubated with fluorescently labeled streptavidin. Excess streptavidin is removed after incubation. (Setup C) To designate both the sequence-dependence and the dependence on the monovalent salt concentration of the vesicle self-assembly process two VPs either loaded with complementary (VP C1, VP C2) or noncomplementary (VP C3, VP C4) DNA single strands (ssDNA) are unified in solutions distinct in sodium iodide concentration. The streptavidin solutions were individually preincubated with biotin-ssDNA solutions prior to vesicle decoration (see microtubes holding the streptavidin/biotin-ssDNA solutions). After incubation of vesicles excess streptavidin/biotin-ssDNA is removed. DNA hybridization of complementary ssDNA causes accumulation of linkers and of the fluorescence signal (e.i) in the contact area over time (d to e) that is absent for noncomplementary ssDNAs (e.ii). (e.i) DNA-independent crystallization of streptavidin molecules on the surface of vesicles (*) that distributes stresses arising during/after DNA-mediated self-assembly may stabilize the linking system by compensating streptavidin molecules either incompletely equipped with biotin-ssDNA (**) or anchored only partially (***).

In previous work [20], [21], [48], we presented implementations of multicompartment systems and resolved the problem of readjusting vesicle formation/composition as well as of losing specificity by using linkers consisting of biotinylated DNA single strands (biotin-ssDNA) that were anchored by long and flexible phospholipid-grafted biotinylated PEG tethers via streptavidin as a connector. The problem linked to readjusting the vesicle formation procedure and/or vesicle composition was addressed by incorporating invariable and universal anchoring sites into the membrane during vesicle formation (phospholipid-grafted biotinylated PEG tethers). Since specificity is introduced only in a postprocessing step by strictly hydrophilic linkers (biotin-ssDNA linked to streptavidin) the vesicle formation procedure and vesicle composition can be kept uniform. Streptavidin is a tetrameric protein that provides two pairs of biotin-binding sites on opposite sides of each streptavidin molecule and that does not affect vesicle stability even if the surface of vesicles is completely coated with a monomolecular layer of streptavidin [49]. The biotin-streptavidin system offers the strongest non-covalent biological interaction known [50], a multitude of possible vesicle modifications, component modularity, and off-the-shelf availability. Since (i) the DNA strands are anchored by two phospholipid-grafted biotinylated PEG tethers per streptavidin molecule, (ii) the streptavidin crystallizes on the surface of vesicles [49], [51], and (iii) the phospholipid-grafted biotinylated PEG tethers provide high detachment resistance [52] and no detectable intermembrane transfer of linkers from donor liposomes to acceptor liposomes [53] it is reasonable to conclude that loss of specificity described for current DNA-mediated linking mechanisms remains absent for the tethering method presented in this study (see Figure 1, setup C, panel e.i for a schematic representation of factors that stabilize the linking system).

The lateral mobility of linkers results in a linkage-induced receptor accumulation at contact areas of adjacent and complementary vesicles [18], [22], [30], [54]–[58]. The depletion of linkers between the so-called adhesion plaques potentially terminates the (self-)assembly process and therefore defines the spatial arrangement of multi-vesicle aggregates [22], [40]. Thus, multi-vesicle aggregates may outperform hard sphere colloids not only in the ability of controlled confinement, transport, and manipulation of chemical cargo but also in the controllability of spatial organization by inherent material properties.

In the present study, we systematically analyzed (see Figure 1 for a detailed description) the single components of the DNA-based linking system by investigating the incorporation of phospholipid-grafted biotinylated PEG tethers to the vesicle membrane during vesicle formation (setup A), the streptavidin loading of the vesicle membrane in dependence of anchor sites concentration (setup B), and the specificity of the DNA-mediated vesicle-vesicle self-assembly in dependence of sequence complementarity and monovalent salt concentration (setup C). We subsequently discuss how multi-vesicle assemblies of predefined architecture may affect analysis, synthesis, and personalized medicine.

## Results and Discussion

### Setup A: Incorporation of phospholipid-grafted biotinylated PEG tethers to the vesicle membrane

**Figure 2:**
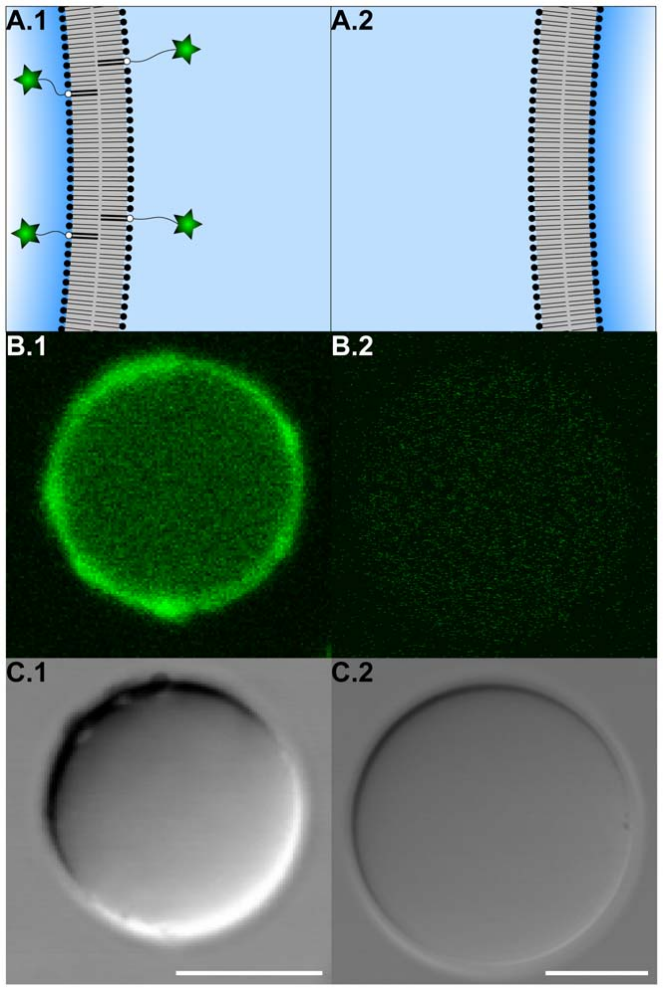
Experimental setup A: Incorporation of phospholipid-grafted biotinylated PEG tethers to the vesicle membrane. (A) Schematic representation of the result of experimental setup A (cp. Figure 1, setup A, panels a.i and a.ii). (B) Confocal laser scanning fluorescence and (C) differential interference contrast micrographs of vesicle population (VP) A1 (left) and A2 (right). For a detailed description of the experimental setup see Figure 1 (setup A). VPs differed in the presence (VP A1) and absence (VP A2) of phospholipid-grafted fluorescently labeled PEG tethers (carboxyfluorescein, pseudocolored green in fluorescence micrographs) during vesicle formation. Scale bars represent 10 µm.

For microscopic analysis of the incorporation of phospholipid-grafted biotinylated PEG tethers to the vesicle membrane, biotin labeled PEG phospholipids (bPEG2000-DSPE) were replaced by carboxyfluorescein labeled PEG phospholipids (cfPEG2000-DSPE). A fluorescence signal was found exclusively at the vesicle membrane and only if fluorescently labeled phospholipids were present during vesicle formation (Figure 2). Thus, phospholipid-grafted biotinylated PEG tethers are incorporated into the vesicle membrane if present during vesicle formation.

### Setup B: Streptavidin loading of the vesicle membrane in dependence of anchor sites concentration

**Figure 3:**
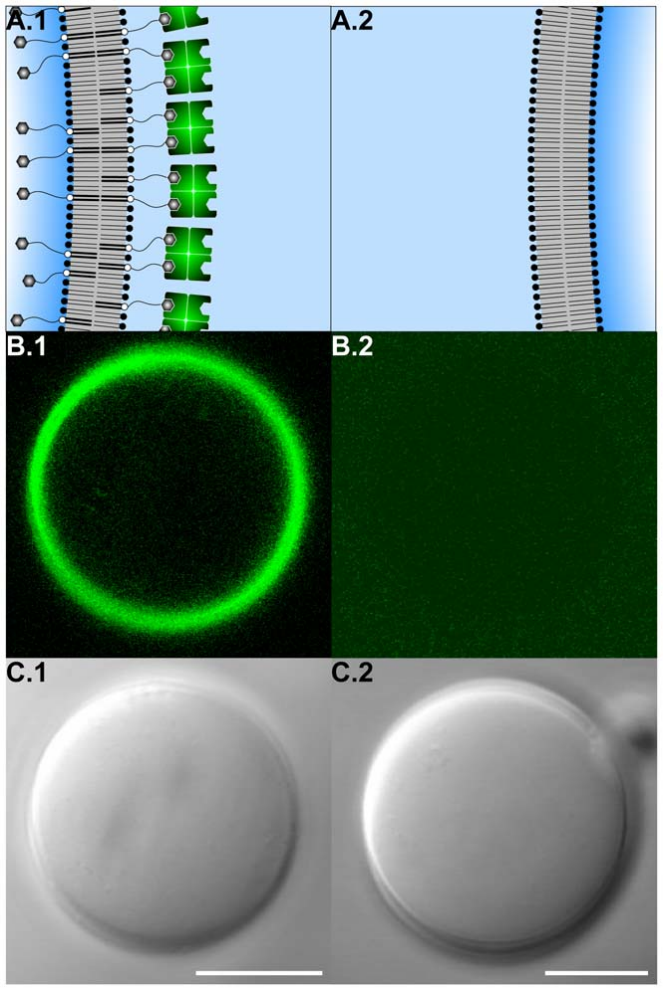
Experimental setup B: Streptavidin loading of the vesicle membrane in dependence of anchor sites concentration. (A) Schematic representation of the result of experimental setup B (cp. Figure 1, setup B, panels c.i and c.ii). (B) Confocal laser scanning fluorescence and (C) differential interference contrast micrographs of vesicle population (VP) B1 (left) and B2 (right). For a detailed description of the experimental procedure see Figure 1 (setup B). VPs differed in the presence and absence of phospholipid-grafted biotinylated PEG tethers during vesicle formation. Both VPs were subsequently incubated with an excess of fluorescently labeled streptavidin (Alexa Fluor® 488, pseudocolored green in fluorescence micrographs). Scale bars represent 10 µm.

Decoration of vesicle surfaces with fluorescently labeled streptavidin can be observed exclusively if anchoring sites – provided by bPEG2000-DSPE – were incorporated into the membrane during vesicle formation (Figure 3). Thus, specificity of decoration is provided by the presence of anchoring sites.

### Setup C: Implementation of a DNA-mediated vesicle-vesicle self-assembly

**Figure 4:**
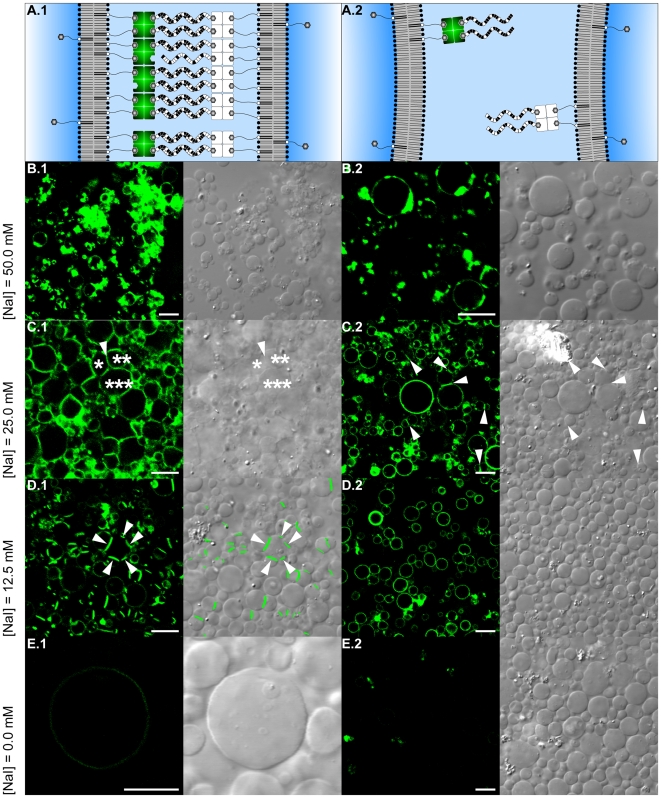
Experimental setup C: Implementation of a DNA-mediated vesicle-vesicle self-assembly. (A) Schematic representation of the result of experimental setup C (cp. Figure 1, setup C, panels e.i and e.ii). (B–E) Confocal laser scanning fluorescence (left) and differential interference contrast (right) micrographs in two columns of merged vesicle populations (VPs) C1 to C4 (VP C1 & VP C2, VP C3 & VP C4). For a detailed description of the experimental procedure see Figure 1 (setup C). The biotinylated membranes (receptor surface density: 1.0 mol % biotin labeled PEG phospholipids) of all VPs were loaded with biotinylated single stranded DNA (biotin-ssDNA) using streptavidin as a cross-linking agent. VPs differed in streptavidin labeling (VPs C1/3: Alexa Fluor® 488, pseudocolored green in fluorescence micrographs, VPs C2/4: unlabeled) and biotin-ssDNA sequence (VP C1/3: α, VP C2: α', VP C4: β') – only sequences α and α' were complementary. Row headings indicate sodium iodide concentrations in the vesicle lumen and the surrounding medium. Fluorescence labeling of the membranes, silhouette blurring indicating vesicle lysis (cp. B.1, B.2), and accumulation of fluorescence signal positively correlate with sodium iodide concentration (microscope settings were identical for all pictures) causing a tradeoff between membrane loading, DNA hybridization, and vesicle stability. Adhesion plaques indicate stable vesicle-vesicle-linkage (visible adhesion plaques are highlighted in the differential interference contrast micrograph of D.1 by an image overlay with the confocal laser scanning fluorescence (processed) micrograph). The adhesion plaques of one vesicle (D.1) and DNA-independent vesicle-vesicle linkages (C.2) are highlighted by arrows. See text for a discussion of the loss of specificity of the DNA-mediated adhesion process observed in panels C.1 and C.2. Panel D.1 is reproduced with kind permission of Springer Science+Business Media (for original publication see [21]). Scale bars represent 10 µm.

Adhesion plaques (cp. Figure 4.D.1) emerging from DNA hybridization were found exclusively if DNA strands were complementary and sodium iodide was present at a concentration of 12.5 millimolar. At monovalent salt concentrations above 12.5 millimolar silhouette blurring (Figure 4.B.1, B.2) and a lower number of vesicles indicate reduced vesicle stability (lysis). Furthermore, DNA-independent vesicle-vesicle-linkage (Figure 4.C.2 (arrows)) as well as the emergence of a homogenous layer of interconnected vesicles (Figure 4.C.1) both indicate a loss of specificity of the adhesion process. Specificity therefore negatively correlates with monovalent salt concentration in the surrounding medium. In particular for two vesicles (*, ** in Figure 4.C.1) to be linked to a third one (***), vesicles * and ** have to present DNA strands of the same sequence on their surface theoretically inhibit their mutual linkage yet observed in Figure 4.C.1 (arrow). At a monovalent salt concentration of 12.5 millimolar, the formation of adhesion plaques depended on the complementarity of the DNA single strands (cp. Figure 4.D.1 vs. Figure 4.D.2) indicating specificity and hence programmability of the DNA-mediated self-assembly process. Since accumulation of fluorescently labeled streptavidin was absent if the DNA strands were not complementary (cp. Figure 4.D.2), both DNA-independent vesicle aggregation mediated by streptavidin [29] and linkage-independent crystallization of streptavidin [59] can be ignored as factor of adhesion plaque formation. Since a streptavidin molecule offers two pairs of biotin-binding sites, streptavidin and biotin-ssDNA concentrations were kept at a molar ratio of 1∶2 during preincubation (prior to vesicle decoration, see Figure 1, setup C) to ensure that on average two binding sites were kept clear in order to link the streptavidin to the vesicle membranes. The absence of a DNA-independent vesicle aggregation mediated by streptavidin may be explained by the absence of free phospholipid-grafted biotinylated PEG tethers on the surface of the vesicle membranes after incubation with streptavidin. Fluorescence intensity was found to correlate positively with monovalent salt concentration in the surrounding medium. This dependence of binding efficiency of biotin-streptavidin on the concentration of sodium iodide is consistent with enhanced binding [60] and reduced dissociation efficiency [61] of streptavidin-biotin in the presence of mono- and divalent salts. An acceptable compromise between the binding efficiency of biotin-streptavidin and the specificity of the adhesion process was found in a salt concentration of 12.5 millimolar sodium iodide. In preliminary experiments (data not shown) vesicle aggregation was unspecific to a lesser extent and vesicles were more stable if sodium iodide was used instead of sodium chloride that is widely applied in vesicle self-assembly experiments. Due to the dependence of biotin-streptavidin binding on monovalent salt concentration, the well-known dependence of DNA hybridization on monovalent salt concentration [62], [63] could not be evaluated accurately herein. Since fluorescently labeled and unlabeled vesicles occurred approximately in equal numbers (cp. Figure 4.D.2) one can conclude that no transfer of linkers between the membranes of different vesicles occurred during experimentation (cp. [40], [47]). In the absence of monovalent ions, differences in fluorescence intensity between setups B and C (cp. Figure 3.B.1 vs. Figure 4.E.1) can be attributed to differences in the relative number of anchoring sites and distinct microscopic settings optimized to detect the weak fluorescence signal in Figure 3.B.1.

**Figure 5:**
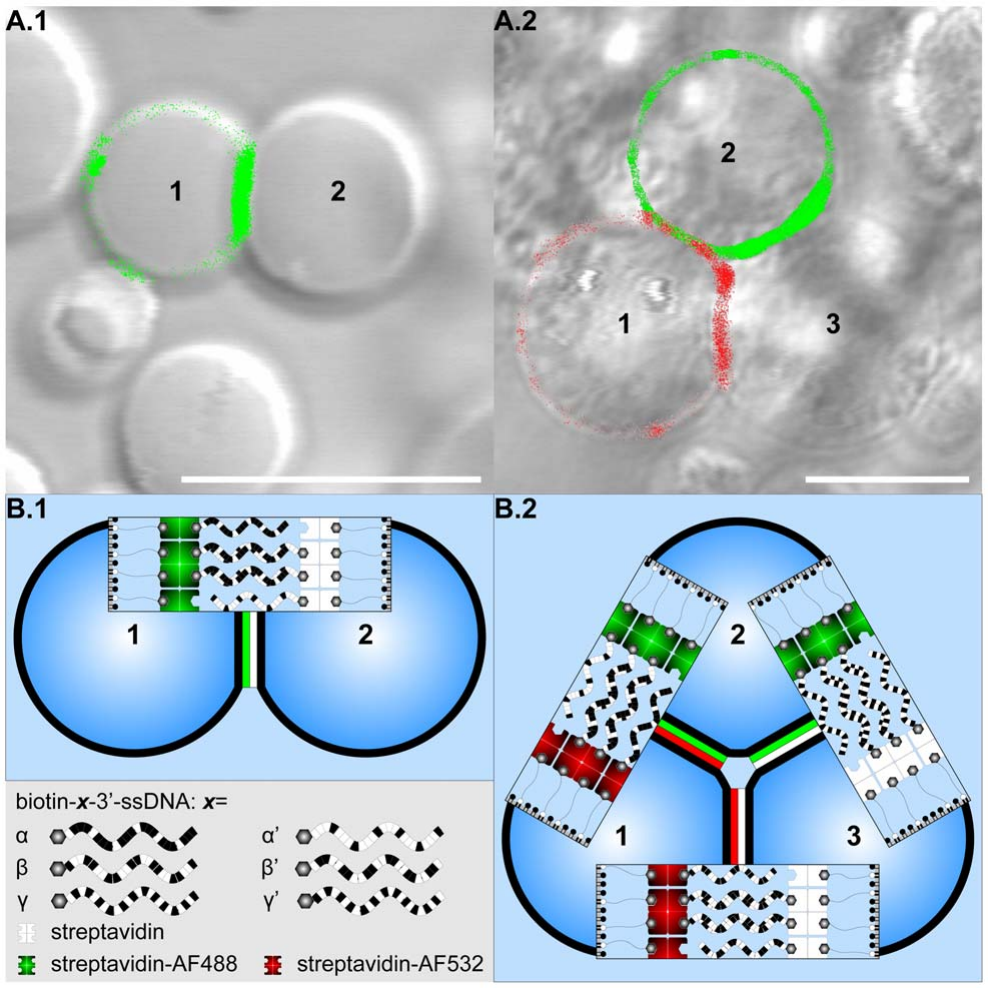
Programmability of the DNA-mediated self-assembly process: Multi-vesicle assemblies of predefined architecture. (A) Image overlays of confocal laser scanning fluorescence and differential interference contrast micrographs of merged vesicle populations (VPs). Biotinylated DNA single strands (biotin-ssDNA) that differ in sequence (α, α', β, β', γ, γ') and streptavidin populations that differ in fluorescence labeling (unlabeled (st.), Alexa Fluor® 488 labeled (st.-AF488, pseudocolored green in fluorescence micrographs), Alexa Fluor® 532 labeled (st.-AF532, pseudocolored red in fluorescence micrographs)) were incubated pairwise prior to vesicle decoration resulting in the following streptavidin/biotin-ssDNA combinations decorating the VPs: (A.1) α-st.-AF488: VP1, α'-st.:VP2; (A.2) α-st.-AF532 & β-st.-AF532: VP1, α'-st.-AF488 & γ-st.-AF488: VP2, β'-st. & γ'-st.: VP3. The receptor surface density was reduced to 0.25 mol % biotin labeled PEG phospholipids (cp. 1.0 mol % in experimental setup C). The fluorescence signal accumulates in the contact areas of adjacent and complementary vesicles forming adhesion plaques that indicate stable vesicle-vesicle linkage. (B) Schematic representation of the programmability of the DNA-mediated self-assembly process. The formation of adhesion plaques depends on the complementarity of ssDNA (cp. Figure 4.C.1) resulting in a sequence depend accumulation of linkers in the contact areas. The depletion of linkers in between the adhesion plaques terminates the self-assembly process. In combination with the ssDNA decoration of the vesicle surface, the self-termination defines the spatial arrangement of multi-vesicle aggregates. Thus, control of the assembly process is inherent to the system resulting either in duplets (A.1) or triplets (A.2) as minimal self-containing structural units. For a discussion of factors causing the low number of such units see text. Scale bars represent 10 µm.

Theoretical predictions assume total streptavidin vesicle surface coverage and, as a consequence, absence of linker depletion between adhesion plaques at a receptor surface density of 0.80 mol % bPEG2000-DSPE [52]. Linker accumulation at 1.00 mol % bPEG2000-DSPE (Figure 4.D.1) indicates incomplete streptavidin coverage and is consistent with the experimental data shown in Figure 5b of [52]. Moreover, linker surface coverage is high enough for single vesicles to form several adhesion plaques (see arrows in Figure 4.D.1). Linkage-induced receptor accumulation [30], [54]–[56] is of particular interest in vesicle self-assembly due to its potential to self-terminate the assembly process by linker depletion and therefore to determine the coordination number of vesicles [29] in dependence of surface linker density [18], [57], [58]. Reducing the surface receptor density to 0.25 mol % bPEG2000-DSPE and increasing the number of distinct populations of complementary DNA strands decorating vesicles resulted in a small number of multi-vesicle structures of gradually increasing aggregate complexity (Figure 5). A detailed description of the experimental procedure is offered in [20]. However, the absolute number of linkers on the vesicle surface not only depends on the fraction of anchoring sites but also on the membrane area. In order to provide an effective self-terminating self-assembly process, vesicle size distribution may have to be monodisperse in addition to the constant surface linker density in future studies to ensure equal numbers of complementary linkers on the vesicle surfaces.

Multi-vesicle assemblies of predefined architecture (cp. Figure 5) may affect at least three distinct domains. (i) Analysis. Starting with a minimal system, the complexity of a bottom-up system may be gradually increased facilitating the understanding of the components and their interaction. Current bottom-up model systems in the analysis of biological processes are restricted to single unilamellar vesicles [3]–[5]. In this way, multi-vesicle assemblies provide an artificial bottom-up model system that allows to emulate and analyze natural cell-cell communication for instance (see (ii) for a scenario how to implement vesicle-vesicle communication in multi-vesicle assemblies). (ii) Synthesis. Externally triggered gating and channeling of confined cargo have already been described for a nanofluidic system which consists of two populations of nanometer-sized vesicles that are enclosed at random in a larger vesicle and consecutively release their attoliter volumes into the larger vesicle which serves as reaction vessel [13]. It has been proposed that communication within a DNA-mediated aggregate would become programmable and more reliable if its adhesion plaques differed in their phospholipid composition resulting in a multicompartment communication network of programmable architecture (see [48] for a discussion). Since this method could improve reliability, versatility and handling, such networks could potentially outperform current single or multicompartment bioreactors. (iii) Personalized medicine. Many therapeutic drugs have undesirable properties that constitute barriers in clinical drug application. Single vesicles are admittedly and successfully used as pharmaceutical carriers targeting active drugs to the site of action (see [10] for a comprehensive review). However, therapeutically effective multicompartment transports containing a pharmacologically inert prodrug [64] spatially separated from a converter enabling its transformation to an active drug molecule are currently unavailable. In this view, multi-vesicle assemblies such as those analyzed here offer such possibilities and thus represent a significant step in modern pharmacology (see [20] for a discussion).

## Materials and Methods

### Vesicle formation (Figure 1 step 0)

Setups A, B, C: For a schematic illustration of the vesicle formation technique and technical terms used see [65] Figure 1. For modifications see [20], [21], [48]. The main aspects of modification recapitulated briefly: introduction of microplates to increase procedural manageability in laboratory experimentation and introduction of a density difference between the *inter-* and *intra*vesicular solutions to detach the vesicles from the interface between ‘intermediate’ and ‘aqueous phase’. The ‘emulsion phase’ was prepared from sucrose or sucrose and sodium iodide (NaI), and the phospholipids POPC (2-Oleoyl-1-palmitoyl-*sn*-glycero-3-phosphocholine, Sigma-Aldrich, Buchs, Switzerland), bPEG2000-DSPE (1,2-Distearoyl-sn-Glycero-3-Phosphoethanolamine-N-[Biotinyl(Polyethylene Glycol)2000] (Ammonium Salt), Avanti Polar Lipids, Alabaster, AL), and cfPEG2000-DSPE (1,2-distearoyl-sn-glycero-3-phosphoethanolamine-N-[poly(ethylene glycol)2000-N'-carboxyfluorescein] (ammonium salt), Avanti Polar Lipids, Alabaster, AL) that were solved in mineral oil (light, Sigma-Aldrich, Buchs, Switzerland) to a final concentration of 200 µM. POPC was purchased as powder and dissolved in chloroform to a total concentration of 5 mg/ml upon arrival. bPEG2000-DSPE and cfPEG2000-DSPE were purchased as 99% pure chloroform stock solutions (10 mg/ml) and used without further purification. The phospholipids dissolved in chloroform were kept at −20°C until use. After chloroform evaporation (under vacuum, 60 min), addition of mineral oil, sonication (30 min), and overnight incubation at room temperature phospholipid solutions were used within several days. The phospholipid solutions were VP A1: 99 mol % POPC, 1 mol % cfPEG2000-DSPE, VP A2: 100 mol % POPC, VP B1: 90 mol % POPC, VP B2: 10 mol % bPEG2000-DSPE, and VP C1-C4: 99 mol % POPC, 1 mol % bPEG2000-DSPE. The same phospholipid solution was used to produce the ‘intermediate phase’ and the ‘emulsion phase’. The ‘aqueous phase’ was prepared from 1000 mOsm glucose (setups A, B) and NaI (setup C, to a total osmolality of 1000 mOsm, for NaI concentrations see Figure 4). The water-in-oil emulsion of the ‘emulsion phase’ was equiosmolar to the ‘aqueous phase’ and was prepared in microtubes by adding 20 µl, 1000 mOsm sucrose (setups A, B) or sucrose/NaI (setup C, to a total osmolality of 1000 mOsm) solution to 1 ml phospholipid solution. The mixture was mechanically agitated, sonicated three times for five seconds and placed over the ‘intermediate phase’ (100 µl, placed over 100 µl ‘aqueous phase’). After incubation (10 min, room temperature), centrifugation (1500×g, 15 min, 4°C) induced vesicle formation and pelletization in the centre of the well.

### Vesicle decoration (Figure 1 steps 1, 2)

Setups B, C: Streptavidin (Sigma-Aldrich, Buchs, Switzerland) and streptavidin-AF488 (streptavidin, Alexa Fluor® 488 conjugate, Invitrogen, Basel, Switzerland) were dissolved in high quality water (Milli-Q, Millipore, Brussels, Belgium) upon arrival to a final concentration of 0.1 mg/ml. DNA single stranded oligonucleotides with biotin modification were synthesized, purified by HPLC, and dissolved (100 µM) by Sigma-Genosys (Buchs, Switzerland). The oligonucleotides sequences were biotin-TGTACGTCACAACTA-3′ (biotin-α-3′-ssDNA), biotin-TAGTTGTGACGTACA-3′ (biotin-α'-3′-ssDNA), and biotin-TGGAGGGCTCTTTCT-3′ (biotin-β'-3′-ssDNA). The streptavidin solutions were either used directly (setup B), or streptavidins were redissolved (after evaporation) in glucose/NaI solution (setup C) to a final concentration of 333 nM, combined (1∶1, v/v) with biotin-ssDNA solutions (666 nM), and individually incubated for 30 min at room temperature to provide monohomophilic oligonucleotide loading of streptavidin (streptavidin-AF488/biotin-α-3′-ssDNA, streptavidin/biotin-α'-3′-ssDNA, streptavidin/biotin-β'-3′-ssDNA). After aspirating the oil by vacuum, the vesicles were decorated with oligonucleotides. In setup B 90 µl of two vesicle populations were incubated (two hours, room temperature) with 10 µl streptavidin solution. In setup C, four vesicle populations were individually incubated (30 min, room temperature) with loaded streptavidin (two times streptavidin-AF488/biotin-α-3′-ssDNA, streptavidin/biotin-α'-3′-ssDNA, streptavidin/biotin-β'-3′-ssDNA). incubation (30 min). Excess streptavidin and oligonucleotides were removed by the following washing procedure (repeated three times): Pelletization (centrifugation at 1500×g, 10 min, 4°C), removal of supernatant (150 µl) and addition of 150 µl ‘aqueous phase’.

### Self-assembly of multi-vesicle structures (Figure 1 step 3)

Setup C: After pooling (streptavidin-AF488/biotin-α-3′-ssDNA & streptavidin/biotin-α'-3′-ssDNA, streptavidin-AF488/biotin-α-3′-ssDNA & streptavidin/biotin-β'-3′-ssDNA) and pelletization (centrifugation at 1500×g, 10 min, 4°C) of vesicle populations, vesicle aggregates were inspected by confocal laser scanning microscopy. Surface linker density is represented by the fluorescence signal of streptavidin-AF488. Inhomogeneities in the signal of fluorescently labeled streptavidin (accumulation *intra* and depletion *inter* contact areas) indicated formation of adhesion plaques. Presence of adhesion plaques qualified vesicle aggregates as assembled [30].

### Surface modification

To prevent vesicles from adhering to surfaces, 96-well microtiter plates U96 (Thermo Fisher Scientific, Langenselbold, Germany), microscope slides and cover glasses were specifically treated. Incubation steps (100 µl, 10 min, room temperature) were interrupted and followed by washing steps using deionized water of (i) microplates U96: 100 µl Repel Silane (GE Healthcare Europe GmbH, Otelfingen, Switzerland), 100 µl coating solution (10 mg/ml DNA from salmon sperm; Sigma-Aldrich, Buchs, Switzerland), 10 mg/ml BSA (in 1× PBS buffer; Roche Diagnostics GmbH, Mannheim); (ii) microtubes: 200 µl Repel Silane (vortexed several times during incubation to ensure total surface coverage); (iii) microscope slides and cover glasses: Repel Silane (total surface coverage). All surfaces were finally blown dry using compressed air. Observation chambers (area: 44×10 mm) for CLSM were made of Repel Silane treated microscope slides and cover glasses spaced to a distance of about 1 mm.

### Microscopy

An inverted Leica Confocal DMR IRE2 SP2 microscope (Leica Lazer Technik, Heidelberg, Germany) equipped with a Zeiss HCX Apochromat 40.0×, 1.25-numerical-aperture oil immersion lens and Melles Griot argon laser (λ_ex_  = 488 nm) was used for Confocal Laser Scanning Microscopy. cfPEG2000-DSPE and streptavidin-AF488 were excited by the argon laser passing a TD 488/543/633 (setup A) or RSP 500 (setups B, C) excitation beam splitter. The epifluorescence was converted into a static beam by an x-y scanner device, passed a band-pass filter 508/31 (setup A), 507/24 (setup B), 509/20 (setup C) and was focused onto a photomultiplier (PMT fluorescence signal offset/HV settings: -1.7/712.2 (setup A), -11.3/793.7 (setup B), -92.1/672.0).
